# Mott’s Velpeau’s Operative Surgery

**Published:** 1847-06

**Authors:** 


*Mott's Velpeau's Operative Surgery.*—We beg pardon of our friends, the *Woods*, for the inadvertency referred to in the following note, which, by way of correction, we cheerfully insert. We trust that they will reap substantial fruits from the enterprize of the publication, commensurate with the credit which belongs to them for completing it in so satisfactory a manner.

New-York, 5th Month, 29, 1847.

To the Editor of the Buffalo Med. Journal:

Having recently completed Mott's Velpeau's Op. Surgery, by publishing the 3d Vol. and Atlas, we sent a copy by our mutual friend, Dr. Lee, to be noticed in the Journal. In the No. of the Journal for this month, we observe that credit is given for the publication to *H. G. Langley*. This may bring the work before the Profession, but publishers do not like that others should reap the credit of their enterprize. We are now the publishers of the whole work, and shall feel obliged for a continuance of the favorable regard which the Medical Press has thus far extended to it.

Respectfully, S. S. & W. WOOD.

